# Supplementary material for: Using both qualitative and quantitative data in parameter identification for systems biology models
Source: Nat Commun. 2018 Sep 25;9:3901. doi: 10.1038/s41467-018-06439-z (PMC6156341; doi:10.1038/s41467-018-06439-z)
Supplement: Supplementary file 1 — Supplementary Information [file 41467_2018_6439_MOESM1_ESM.pdf]

Supplementary Information

Using both qualitative and quantitative data in parameter identification for systems biology models

Eshan D. Mitra, Raquel Dias, Richard G. Posner, and William S. Hlavacek

Alpha factor dataset

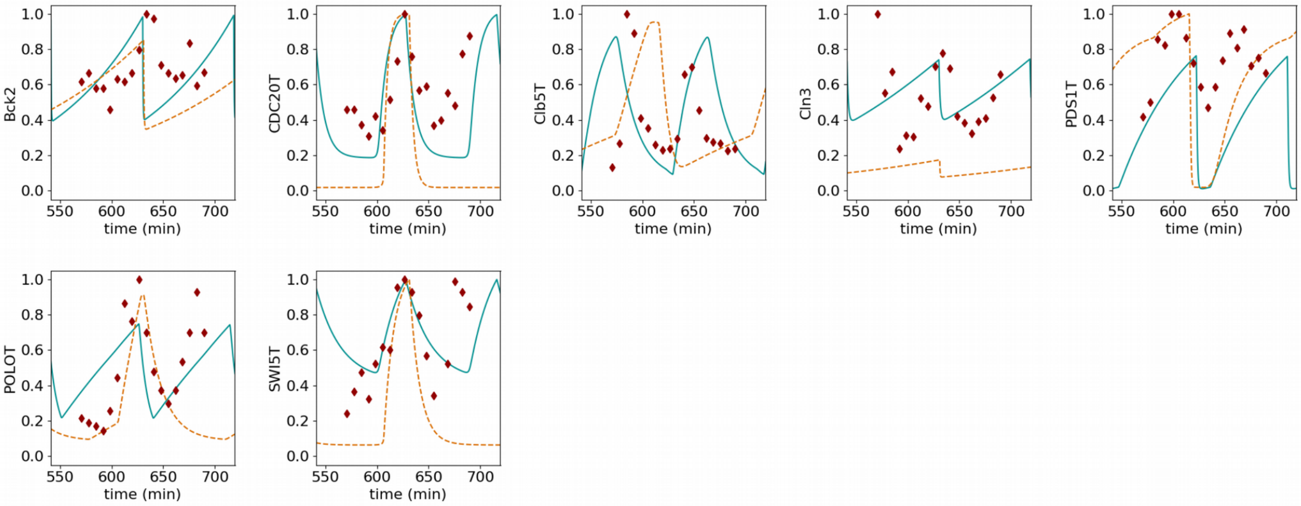

cdc15-ts **dataset**

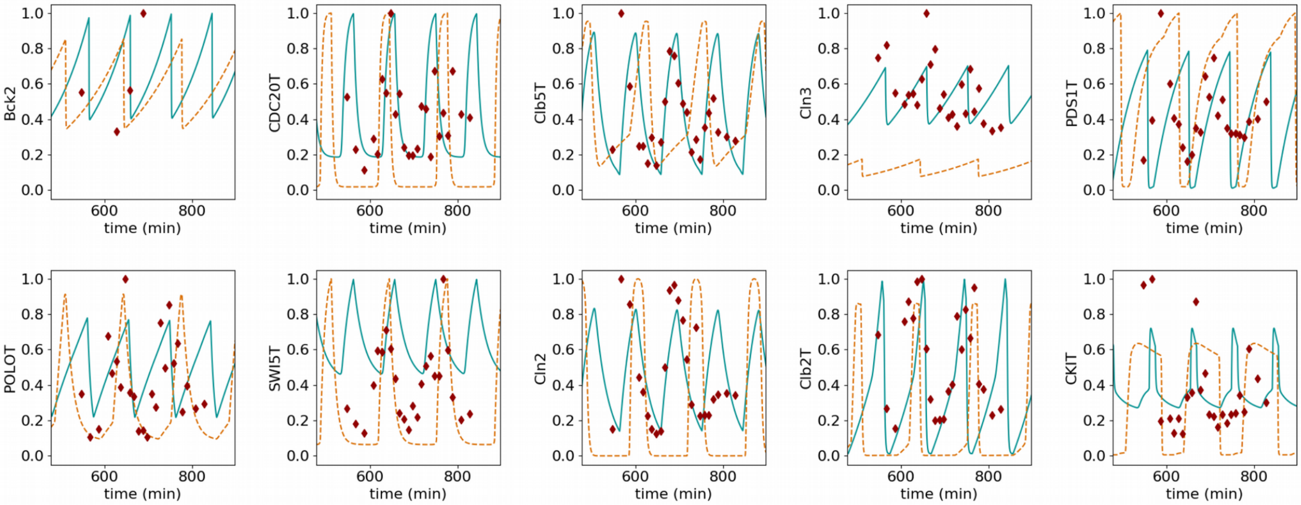

cdc28-ts **dataset**

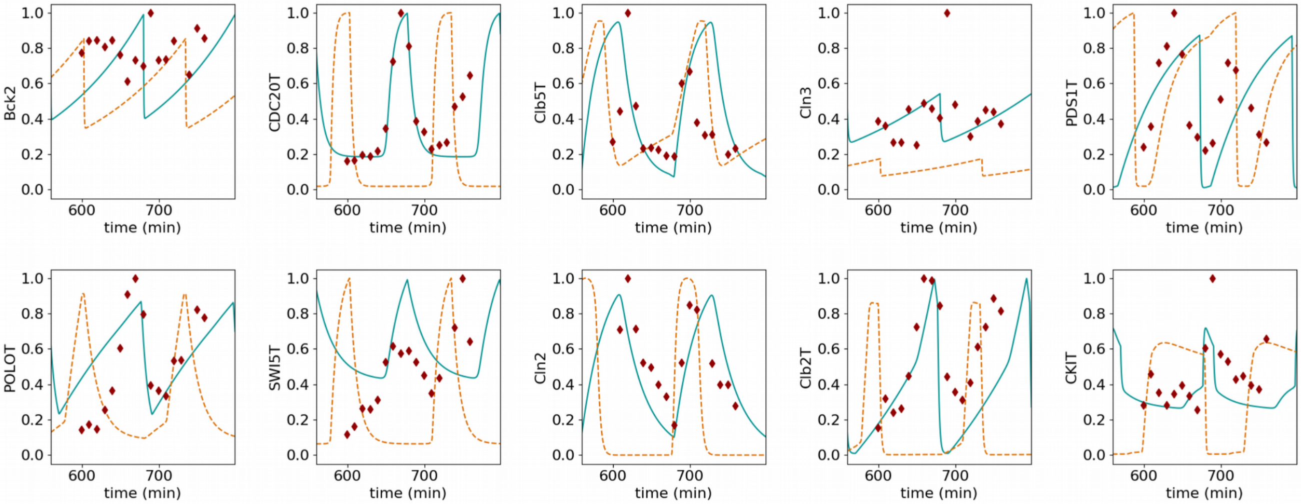

Supplementary Figure 1: Additional quantitative data and resulting fits for the yeast cell cycle. Red diamonds show the experimental data from ref. [1] Solid blue curves give results from our best fit. Dashed orange curves give results for the best fit of ref. [2], which was not fit to quantitative data. Three datasets from ref. [1] are shown, in which a population of cells was synchronized with alpha factor, a *cdc15-ts* mutation, or a *cdc28-ts* mutation, respectively.

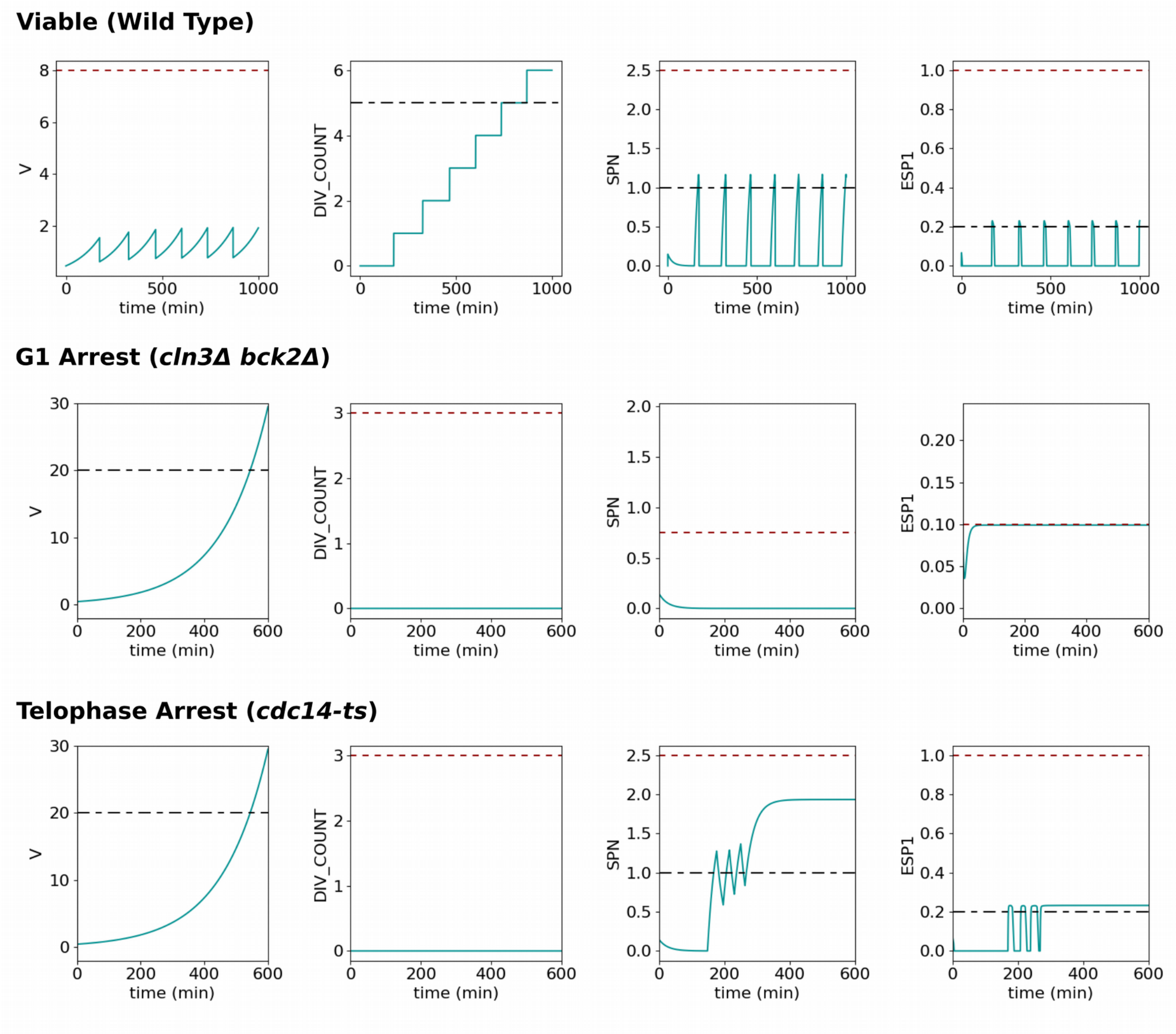

Supplementary Figure 2: Additional time traces of model outputs subject to qualitative constraints. Black dash-dot lines indicate a value that the trace must reach; red dashed lines indicate a value that the trace must not reach.

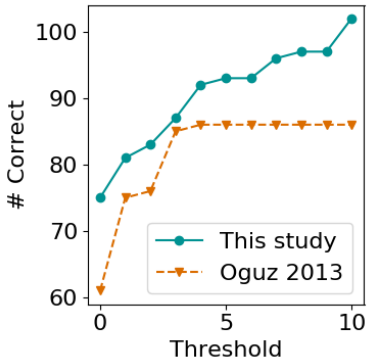

Supplementary Figure 3: Comparison of our best fit to that of Oguz et al. 2013 (ref. [2]), in terms of the number of mutant strains simulated correctly according to our constraints. A mutant is scored as correctly simulated if the total penalty function value is no more than the "Threshold" indicated on the x coordinate. Note that a similar number of correct predictions (e.g. ~ 85 at Threshold = 4) does not imply that the same 85 mutants were predicted correctly in each case.

If we evaluate according to the metric used in ref. [2] – scoring each mutant as viable or inviable based on the shape of the *V* trace – our fit is consistent with 96 mutants, ref. [2] is consistent with 106. This difference is not surprising, given that our implementation is additionally fitting to quantitative data and data on the phase of cell cycle arrest.

Supplementary Table 1: Qualitative constraints used in fitting of the yeast cell cycle model

System Property	Constraint	Weight, C_i*
Constraints for viable mutants		
Cell divides before volume V reaches 8	$\max(V(t)) < 8$	0.005
Cell completes at least 5 divisions	$\max(\text{DIV\_COUNT}(t)) > 5$	3
Origin activation ORI is completed	$\max(\text{ORI}(t)) > 5$	2
Spindle activation SPN is completed	$\max(\text{SPN}(t)) > 1$	10
ESP1 is activated	$\max(\text{ESP1}(t)) > 0.2$	20
ORI has oscillatory behavior †‡	$\max(\text{ORI}(t)) < 40$	0.01
SPN has oscillatory behavior †	$\max(\text{SPN}(t)) < 2$	10
ESP1 does not increase without bound	$\max(\text{ESP1}(t)) < 1$	10
Bud forms before spindle assembly completes	$\text{FLAG\_BUD}(\tau) \geq 1$ $\tau\text{: FLAG\_SPC}(\tau) = 1$	$\min(1, 10 * (1 - BUD(\tau)))$
Origin activation completes before spindle assembly completes	$\text{FLAG\_UDNA}(\tau) \geq 1$ $\tau\text{: FLAG\_SPC}(\tau) = 1$	$\min(1, 10 * (1 - \text{ORI}(\tau)))$
A bud forms before each division	$\text{FLAG\_BUD}(\tau_1) \geq 1$ $\tau_1\text{: DIV\_COUNT}(\tau_1) = 1$	$\min(1, 10 * (1 - BUD(\tau_1)))$
	$\text{FLAG\_BUD}(\tau_2) \geq 1$ $\tau_2\text{: DIV\_COUNT}(\tau_2) = 2$	$\min(1, 10 * (1 - BUD(\tau_2)))$
	$\text{FLAG\_BUD}(\tau_3) \geq 1$ $\tau_3\text{: DIV\_COUNT}(\tau_3) = 3$	$\min(1, 10 * (1 - BUD(\tau_3)))$
Origin activation occurs before each division, and origin relicensing occurs in each cell cycle after the first	$\text{FLAG\_UDNA}(\tau_1) \geq 1$ $\tau_1\text{: DIV\_COUNT}(\tau_1) = 1$	$\min(1, 10 * (1 - \text{ORI}(\tau_1)))$
	$\text{FLAG\_UDNA}(\tau_2) \geq 1$ $\tau_2\text{: DIV\_COUNT}(\tau_2) = 2$	$\min(1, 10 * (1 - \text{ORI}(\tau_2)))$
	$\text{FLAG\_UDNA}(\tau_3) \geq 1$ $\tau_3\text{: DIV\_COUNT}(\tau_3) = 3$	$\min(1, 10 * (1 - \text{ORI}(\tau_3)))$
Spindle assembly occurs before each division	$\text{FLAG\_SPC}(\tau_1) \geq 1$ $\tau_1\text{: DIV\_COUNT}(\tau_1) = 1$	$\min(1, 10 * (1 - \text{SPN}(\tau_1)))$
	$\text{FLAG\_SPC}(\tau_2) \geq 1$ $\tau_2\text{: DIV\_COUNT}(\tau_2) = 2$	$\min(1, 10 * (1 - \text{SPN}(\tau_2)))$
	$\text{FLAG\_SPC}(\tau_3) \geq 1$ $\tau_3\text{: DIV\_COUNT}(\tau_3) = 3$	$\min(1, 10 * (1 - \text{SPN}(\tau_3)))$
Constraints for G1 arrest mutants		
Cell volume V increases without bound	$\max(V(t)) > 20$	2
Cell cycle arrests before 3 divisions are completed	$\max(\text{DIV\_COUNT}(t)) < 3$	5
Origin activation ORI does not complete while the cell is alive ‡	$\max_{t \in [0,\tau]\,:\,V(\tau)=8}(\text{ORI}(t)) < 0.75$	2
Spindle assembly SPN does not complete while the cell is alive	$\max_{t \in [0,\tau]\,:\,V(\tau)=8}(\text{SPN}(t)) < 0.75$	10
ESP1 activation does not occur while the cell is alive	$\max_{t \in [0,\tau]\,:\,V(\tau)=8}(\text{ESP1}(t)) < 0.1$	50
SPN does not increase without bound	$\max(\text{SPN}(t)) < 2$	10
ESP1 does not increase without bound	$\max(\text{ESP1}(t)) < 1$	10
Constraints for S/G2 arrest mutants		
Cell volume V increases without bound	$\max(V(t)) > 20$	2
Cell cycle arrests before 3 divisions are completed	$\max(\text{DIV\_COUNT}(t)) < 3$	5
Origin activation ORI is completed while the cell is alive	$\max(\text{ORI}(t)) > 5$	2
	$\text{ORI}(\tau) > 5$ $\tau\,:\,V(\tau) = 8$	2
Spindle assembly SPN does not complete while the cell is alive	$\max_{t \in [0,\tau]\,:\,V(\tau)=8}(\text{SPN}(t)) < 0.75$	10
ESP1 activation does not occur while the cell is alive	$\max_{t \in [0,\tau]\,:\,V(\tau)=8}(\text{ESP1}(t)) < 0.1$	50
SPN does not increase without bound	$\max(\text{SPN}(t)) < 2$	10
ESP1 does not increase without bound	$\max(\text{ESP1}(t)) < 1$	10
Constraints for metaphase arrest mutants		
Cell volume V increases without bound	$\max(V(t)) > 20$	2
Cell cycle arrests before 3 divisions are completed	$\max(\text{DIV\_COUNT}(t)) < 3$	5
Origin activation ORI is completed while the cell is alive	$\max(\text{ORI}(t)) > 5$	2
	$\text{ORI}(\tau) > 5$ $\tau\,:\,V(\tau) = 8$	2
Spindle assembly SPN is completed while the cell is alive	$\max(\text{SPN}(t)) > 1$	10
	$\text{SPN}(\tau) > 1$ $\tau\,:\,V(\tau) = 8$	10
ESP1 activation does not occur while the cell is alive	$\max_{t \in [0,\tau]\,:\,V(\tau)=8}(\text{ESP1}(t)) < 0.1$	50
SPN does not increase without bound	$\max(\text{SPN}(t)) < 2$	10
ESP1 does not increase without bound	$\max(\text{ESP1}(t)) < 1$	10

System Property	Constraint	Weight, C_i*
Constraints for telophase arrest mutants		
Cell volume V increases without bound	$\max(V(t)) > 20$	2
Cell cycle arrests before 3 divisions are completed	$\max(\text{DIV\_COUNT}(t)) < 3$	5
Origin activation ORI is completed while the cell is alive	$\max(\text{ORI}(t)) > 5$	2
	$\text{ORI}(\tau) > 5$ $\tau : V(\tau) = 8$	2
Spindle assembly SPN is completed while the cell is alive	$\max(\text{SPN}(t)) > 1$	10
	$\text{SPN}(\tau) > 1$ $\tau : V(\tau) = 8$	10
ESP1 activation occurs while the cell is alive	$\max(\text{ESP1}(t)) > 0.2$	20
	$\text{ESP1}(\tau) > 0.2$ $\tau : V(\tau) = 8$	20
SPN does not increase without bound	$\max(\text{SPN}(t)) < 2$	10
ESP1 does not increase without bound	$\max(\text{ESP1}(t)) < 1$	10
Constraints for mutants with origin relicensing problems		
Cell completes at least 5 divisions	$\max(\text{DIV\_COUNT}(t)) > 5$	3
Origin activation ORI is completed	$\max(\text{ORI}(t)) > 5$	2
Spindle assembly SPN is completed	$\max(\text{SPN}(t)) > 1$	10
ESP1 is activated	$\max(\text{ESP1}(t)) > 0.2$	20
ORI does not increase without bound	$\max(\text{ORI}(t)) < 40$	0.01
CLB2 + CLB5 never drops below 0.25 following the first division	$\min(\text{CLB2}(t) + \text{CLB5}(t)) > 0.25$ $t \in [\tau_1, \tau_2] : \text{DIV\_COUNT}(\tau_1) = 1, \text{DIV\_COUNT}(\tau_2) = 2$	40
SPN does not increase without bound	$\max(\text{SPN}(t)) < 2$	10
ESP1 does not increase without bound	$\max(\text{ESP1}(t)) < 1$	10
Constraints for mitotic catastrophe mutants		
Cell completes at least 5 divisions	$\max(\text{DIV\_COUNT}(t)) > 5$	3
Origin activation ORI is completed while the cell is alive	$\max(\text{ORI}(t)) > 5$	2
	$\text{ORI}(\tau) > 5$ $\tau : V(\tau) = 8$	2
Spindle assembly SPN is completed	$\max(\text{SPN}(t)) > 1$	10
ESP1 is activated before SPN completes	$\text{ESP1}(\tau) > 0.2$ $\tau : \text{SPN}(\tau) = 1$	20
SPN does not increase without bound	$\max(\text{SPN}(t)) < 2$	10
ESP1 does not increase without bound	$\max(\text{ESP1}(t)) < 1$	10
Constraints for inviable mutants (phase of arrest unknown)		
Cell volume V increases without bound	$\max(V(t)) > 20$	2
Cell cycle arrests before 3 divisions are completed	$\max(\text{DIV\_COUNT}(t)) < 3$	5
SPN does not increase without bound	$\max(\text{SPN}(t)) < 2$	10
ESP1 does not increase without bound	$\max(\text{ESP1}(t)) < 1$	10

For constraints in effect for specific time values, τ is defined as the earliest time such that the specified condition is met.

Note that "flag" variables FLAG_BUD, FLAG_UDNA, and FLAG_SPC take values of only 0 or 1, and are used to track the occurrence of certain events in each cell cycle: bud formation, origin activation, and spindle assembly, respectively. The flag variables are set to 0 at each cell division. When the corresponding continuous variable (BUD for FLAG_BUD, ORI for FLAG_UDNA, SPN for FLAG_SPC) reaches 1, indicating completion of the event, the flag variable is set to 1. When constraints based on flag variables are violated, we instead assign a penalty based on the value of the corresponding continuous variable.

* The weights shown were hand-chosen to give each constraint roughly equal influence on the objective function value. When combining with quantitative data, these weights were all divided by a factor of 15 to give roughly equal contributions from the quantitative data and the constraints.

† As a surrogate for oscillatory behavior, which is difficult to express directly with an inequality constraint, we imposed an upper bound on the variable value. This proved effective because the alternative to oscillatory behavior in this model is for the variable to increase without bound. The value of the upper bound was chosen based on our initial exploration of parameter space, such that oscillatory time courses did not typically exceed the bound, but monotonically increasing time courses did typically exceed the bound.

Based on the results shown for the fit of ref. [2] in Supplementary Table 2, our choices of upper bounds for ORI(t) and SPN(t) appear acceptable. A concern would be if ORI(t) or SPN(t) exceeded the bound while still being oscilliatory, and therefore consistent with a viable phenotype. However, in the fit of ref. [2] (which was not optimized to these upper bounds), we see that this particular situation—a penalty for exceeding the ORI(t) or SPN(t) upper bound in an otherwise acceptable viable case—occurs infrequently, and when it does occur, it results in a very small penalty. In Supplementary Table 2, the total penalty resulting from this case is 3.27 (all

from the ORI(t) constraint), with a maximum of 0.51 on a single mutant.

‡ These properties resulted in very high penalties when violated in the fit of ref. [2], which is an undesirable feature of our objective function. However, the weights of the corresponding constraints were necessary to enforce the stated system properties in our fit. If the weights were lower, it would be possible to find parameter sets that violate the system properties but receive low penalties. For example, as written, $\max(\mathrm{ORI}(t)) < 0.75$ would receive a penalty of 0.5 if origin activation completed by reaching exactly 1. If we had reduced the constraint weight by a factor of 10, the resulting penalty of 0.05 would be too small to steer the fit toward satisfying the constraint. Similar reasoning holds for enforcing oscilliatory behavior with the constraint $\max(\mathrm{ORI}(t)) < 40$.

Supplementary Table 2: Yeast mutants used in fitting. For each mutant, we show which constraints were violated and the corresponding penalty function values in our best fit, and the same for the best fit in Oguz et al. 2013 (ref. [2]). Refer to Supplementary Table 1 for a more detailed description of the constraints.

Mutant;<br>Phenotype	Param Changes	[Penalties] Failed Constraints	[Penalties] Failed Constraints<br>(Oguz 2013)
WT<br>Viable	-	-	-
WT in galactose<br>Viable	MDT=150<br>f=0.48	-	-
*cln3*Δ<br>Viable	Dn3=0<br>Cln3=0	-	-
*bck2*Δ<br>Viable	ks_k2=0<br>Bck2=0	-	[0.247] max(ORI(t)) < 40
*cln3*Δ *bck2*Δ<br>G1 Arrest	Dn3=0<br>Cln3=0<br>ks_k2=0<br>Bck2=0	-	[2.4] max(ESP1(t)) < 0.1
*cln3*Δ *bck2*Δ<br>multicopy *CLN2*<br>G1 Arrest	Dn3=0<br>Cln3=0<br>ks_k2=0<br>Bck2=0<br>ks_n2_bf*=2	-	[2.4] max(ESP1(t)) < 0.1
*cln3*Δ *bck2*Δ *sic*Δ<br>G1 Arrest	Dn3=0<br>Cln3=0<br>ks_k2=0<br>Bck2=0<br>ks_ki*=0.125<br>ks_ki_swi5*=0.125<br>CKIT=0.2	[7.81] max($V(t)$) > 20<br>[5] max(DIV_COUNT(t)) < 3<br>[0.0941] max(ORI(t)) < 0.75	[31] max($V(t)$) > 20<br>[15] max(DIV_COUNT(t)) < 3<br>[13.5] max(ESP1(t)) < 0.1<br>[139] max(ORI(t)) < 0.75<br>[16.4] max(SPN(t)) < 0.75
*cln3*Δ *bck2*Δ<br>*whi5*Δ<br>Viable	Dn3=0<br>Cln3=0<br>ks_k2=0<br>Bck2=0<br>WHI5T=0<br>WHI5dep=0	-	-
GAL-CLN3<br>Viable	MDT=150<br>f=0.48<br>Dn3*=20	-	-
Multicopy *BCK2*<br>Viable	ks_k2*=17	-	-
*cln1*Δ *cln2*Δ<br>Viable	ks_n2=0<br>ks_n2_bf=0<br>Cln2=0	[0.0837] max($V(t)$) < 8<br>[3] max(DIV_COUNT(t)) > 5	[0.105] max(ORI(t)) < 40
*cln1*Δ *cln2*Δ *bck2*Δ<br>Viable	ks_n2=0<br>ks_n2_bf=0<br>Cln2=0<br>ks_k2=0<br>Bck2=0	[0.0838] max($V(t)$) < 8<br>[3] max(DIV_COUNT(t)) > 5	[5.49] max($V(t)$) < 8<br>[15] max(DIV_COUNT(t)) > 5<br>[200] max(ORI(t)) < 40
*cln1*Δ *cln2*Δ *sic*Δ<br>Viable	ks_n2=0<br>ks_n2_bf=0<br>Cln2=0<br>ks_ki*=0.125<br>ks_ki_swi5*=0.125<br>CKIT=0.2	-	[4.36] FLAG_BUD(τ) $>= 1$<br>[6.18] FLAG_BUD(τ) $>= 1$<br>[6.01] FLAG_BUD(τ) $>= 1$<br>[6.08] FLAG_BUD(τ) $>= 1$
*cln1*Δ *cln2*Δ *cki*Δ<br>Viable	ks_n2=0<br>ks_n2_bf=0<br>Cln2=0<br>ks_ki=0<br>ks_ki_swi5=0<br>CKIT=0<br>CKIP=0	-	[7.61] FLAG_BUD(τ) $>= 1$<br>[7.62] FLAG_BUD(τ) $>= 1$<br>[8.28] FLAG_BUD(τ) $>= 1$<br>[8.54] FLAG_BUD(τ) $>= 1$

*cln*1Δ *cln*2Δ<br>*GAL-SIC*1<br>G1 Arrest	ks_n2=0<br>ks_n2_bf=0<br>Cln2=0<br>MDT=150<br>f=0.48<br>ks_ki*=33.33	-	[13.5] max(ESP1(t)) $<$ 0.1<br>[300] max(ORI(t)) $<$ 0.75<br>[14.5] max(SPN(t)) $<$ 0.75
*cln*1Δ *cln*2Δ<br>*GAL-CLN*2<br>Viable	ks_n2_bf=0<br>MDT=150<br>f=0.48<br>ks_n2=0.15	-	-
*cln*1Δ *cln*2Δ<br>*GAL-SIC*1<br>*GAL-CLN*2<br>Viable	ks_n2_bf=0<br>MDT=150<br>f=0.48<br>ks_n2=0.15<br>ks_ki*=33.33	[0.0264] max($V(t)$) $<$ 8<br>[9] max(DIV_COUNT(t)) $>$ 5	[0.136] max(ORI(t)) $<$ 40
*cln*1Δ *cln*2Δ<br>*cdh*1Δ<br>Viable	ks_n2=0<br>ks_n2_bf=0<br>Cln2=0<br>CDH1T=0<br>CDH1A=0	[2.31] max($V(t)$) $<$ 8<br>[15] max(DIV_COUNT(t)) $>$ 5<br>[2.27] max(ORI(t)) $<$ 40	[5.49] max($V(t)$) $<$ 8<br>[15] max(DIV_COUNT(t)) $>$ 5<br>[200] max(ORI(t)) $<$ 40<br>[1.97] FLAG_BUD(τ) $>=$ 1
*cln*1Δ *cln*2Δ<br>*cdh*1Δ *GAL-SIC*1<br>G1 Arrest	ks_n2=0<br>ks_n2_bf=0<br>Cln2=0<br>CDH1T=0<br>CDH1A=0<br>MDT=150<br>f=0.48<br>ks_ki*=33.33	-	[13.5] max(ESP1(t)) $<$ 0.1<br>[300] max(ORI(t)) $<$ 0.75<br>[16.2] max(SPN(t)) $<$ 0.75
*cln*1Δ *cln*2Δ<br>*cdh*1Δ *GAL-CLN*2<br>Viable	ks_n2_bf=0<br>CDH1T=0<br>CDH1A=0<br>MDT=150<br>f=0.48<br>ks_n2=0.15	[0.194] max($V(t)$) $<$ 8<br>[15] max(DIV_COUNT(t)) $>$ 5	[3.35] FLAG_BUD(τ) $>=$ 1<br>[4.96] FLAG_BUD(τ) $>=$ 1<br>[6.77] FLAG_BUD(τ) $>=$ 1<br>[6.52] FLAG_BUD(τ) $>=$ 1
*cln*1Δ *cln*2Δ<br>*cdh*1Δ *GAL-SIC*1<br>*GAL-CLN*2<br>Viable	ks_n2_bf=0<br>CDH1T=0<br>CDH1A=0<br>MDT=150<br>f=0.48<br>ks_n2=0.15<br>ks_ki*=33.33	[0.194] max($V(t)$) $<$ 8<br>[15] max(DIV_COUNT(t)) $>$ 5	[0.0158] max(ORI(t)) $<$ 40
*cln*1Δ *cln*2Δ *cln*3Δ<br>G1 Arrest	ks_n2=0<br>ks_n2_bf=0<br>Cln2=0<br>Dn3=0<br>Cln3=0	[5] max(DIV_COUNT(t)) $<$ 3	[5] max(DIV_COUNT(t)) $<$ 3<br>[13.5] max(ESP1(t)) $<$ 0.1<br>[285] max(ORI(t)) $<$ 0.75<br>[16.1] max(SPN(t)) $<$ 0.75
*cln*1Δ *cln*2Δ *cln*3Δ<br>*GAL-CLN*2<br>Viable	ks_n2_bf=0<br>Dn3=0<br>Cln3=0<br>MDT=150<br>f=0.48<br>ks_n2=0.15	-	-
*cln*1Δ *cln*2Δ *cln*3Δ<br>*GAL-CLN*3<br>Viable	ks_n2=0<br>ks_n2_bf=0<br>Cln2=0<br>MDT=150<br>f=0.48<br>Dn3*=20	[0.0616] max($V(t)$) $<$ 8<br>[9] max(DIV_COUNT(t)) $>$ 5	[0.00198] max(ORI(t)) $<$ 40

*cln1*Δ *cln2*Δ *cln3*Δ<br>*sic*Δ<br>Viable	ks_n2=0<br>ks_n2_bf=0<br>Cln2=0<br>Dn3=0<br>Cln3=0<br>ks_ki*=0.125<br>ks_ki_swi5*=0.125<br>CKIT=0.2	-	[5.6] FLAG_BUD(τ) >= 1<br>[6.88] FLAG_BUD(τ) >= 1<br>[7.46] FLAG_BUD(τ) >= 1<br>[7.48] FLAG_BUD(τ) >= 1
*cln1*Δ *cln2*Δ *cln3*Δ<br>multicopy *BCK2*<br>Viable	ks_n2=0<br>ks_n2_bf=0<br>Cln2=0<br>Dn3=0<br>Cln3=0<br>ks_k2*=17	[0.0843] max($V(t)$) $<$ 8<br>[3] max(DIV_COUNT(t)) $>$ 5	[1.17] FLAG_BUD(τ) >= 1
*cln1*Δ *cln2*Δ *cln3*Δ<br>*bck2*Δ *GAL-CLN2*<br>Viable	ks_n2_bf=0<br>Dn3=0<br>Cln2=0<br>ks_k2=0<br>Bck2=0<br>MDT=150<br>f=0.48<br>ks_n2=0.15	-	-
*cln1*Δ *cln2*Δ *cln3*Δ<br>multicopy CLB5<br>Viable	ks_n2=0<br>ks_n2_bf=0<br>Cln2=0<br>Dn3=0<br>Cln3=0<br>ks_b5*=5.33<br>ks_b5_bf*=5.33	[0.00566] max(ORI(t)) $<$ 40	[0.3] max(ORI(t)) $<$ 40
*cln1*Δ *cln2*Δ *cln3*Δ<br>*GAL*-CLB5<br>Viable	ks_n2=0<br>ks_n2_bf=0<br>Cln2=0<br>Dn3=0<br>Cln3=0<br>MDT=150<br>f=0.48<br>ks_b5=0.04	[0.459] max(ORI(t)) $<$ 40<br>[1] FLAG_UDNA(τ) >= 1<br>[1] FLAG_UDNA(τ) >= 1	[0.513] max(ORI(t)) $<$ 40
*cln1*Δ *cln2*Δ *cln3*Δ<br>*GAL*-CLB2<br>G1 Arrest	ks_n2=0<br>ks_n2_bf=0<br>Cln2=0<br>Dn3=0<br>Cln3=0<br>MDT=150<br>f=0.48<br>ks_b2*=49.40	[6.63] max(ESP1(t)) $<$ 0.1<br>[28] max(ORI(t)) $<$ 0.75<br>[11.8] max(SPN(t)) $<$ 0.75	[13.5] max(ESP1(t)) $<$ 0.1<br>[877] max(ORI(t)) $<$ 0.75<br>[16.5] max(SPN(t)) $<$ 0.75
*cln1*Δ *cln2*Δ *cln3*Δ<br>*cdh1*Δ<br>Telophase Arrest	ks_n2=0<br>ks_n2_bf=0<br>Cln2=0<br>Dn3=0<br>Cln3=0<br>CDH1T=0<br>CDH1A=0	[2.58] ORI(τ) $>$ 5	-
*cln2*Δ *cln3*Δ *apc-ts*<br>Metaphase Arrest	ks_n2=0<br>ks_n2_bf=0<br>Cln2=0<br>Dn3=0<br>Cln3=0<br>ks_20=0<br>ks_20_m1=0<br>CDH1T=0<br>CDH1A=0	-	[2.36] max(ESP1(t)) $<$ 0.1

*cdh*1Δ<br>Viable	CDH1T=0<br>CDH1A=0	[2.31] max($V(t)$) < 8<br>[15] max(DIV_COUNT(t)) > 5<br>[2.27] max(ORI(t)) < 40	[4.1] FLAG_BUD(τ) >= 1<br>[9.49] FLAG_BUD(τ) >= 1<br>[8.48] FLAG_BUD(τ) >= 1<br>[8.68] FLAG_BUD(τ) >= 1
*CDH*1<br>consituitively active<br>S/G2 Arrest	CDH1T*=3<br>ki_h1_e=0	-	[2.4] max(ESP1(t)) < 0.1
*sic*1Δ<br>Viable	ks_ki*=0.125<br>ks_ki_swi5*=0.125<br>CKIT=0.2	-	-
*GAL-SIC*1<br>Viable	MDT=150<br>f=0.48<br>ks_ki*=33.33	[0.0264] max($V(t)$) < 8<br>[9] max(DIV_COUNT(t)) > 5	-
*GAL-SIC*1-*db*Δ<br>G1 Arrest	MDT=150<br>f=0.48<br>ks_ki*=33.33<br>kd_kip=0	-	[2.4] max(ESP1(t)) < 0.1
*sic*1Δ *cdc*6Δ *cki*Δ<br>Viable	ks_ki=0<br>ks_ki_swi5=0<br>CKIT=0<br>CKIP=0	-	[7.47] FLAG_BUD(τ) >= 1<br>[7.59] FLAG_BUD(τ) >= 1<br>[8.28] FLAG_BUD(τ) >= 1<br>[8.54] FLAG_BUD(τ) >= 1
*swi*5Δ<br>Viable	ks_swi5=0<br>ks_swi5_m1=0<br>SWI5T=0	-	-
*sic*1Δ *cdc*6Δ2-49<br>*cdh*1Δ<br>Inviable	ks_ki=0<br>ks_ki_swi5=0<br>CKIT=0<br>CKIP=0<br>CDH1T=0<br>CDH1A=0	-	-
*swi*5Δ *cdh*1Δ<br>Inviable	ks_swi5=0<br>ks_swi5_m1=0<br>SWI5T=0<br>CDH1T=0<br>CDH1A=0	-	-
*swi*5Δ *cdh*1Δ<br>*GAL-SIC*1<br>Viable	ks_swi5=0<br>ks_swi5_m1=0<br>SWI5T=0<br>CDH1T=0<br>CDH1A=0<br>MDT=150<br>f=0.48<br>ks_ki*=33.33	-	[3.63] FLAG_BUD(τ) >= 1<br>[6.03] FLAG_BUD(τ) >= 1<br>[6.64] FLAG_BUD(τ) >= 1<br>[6.31] FLAG_BUD(τ) >= 1
*clb*5Δ *clb*6Δ<br>Viable	ks_b5=0<br>ks_b5_bf=0<br>Clb5T=0	[0.901] max($V(t)$) < 8<br>[12] max(DIV_COUNT(t)) > 5	-
*clb*5Δ *clb*6Δ *cln*1Δ<br>*cln*2Δ<br>G1 Arrest	ks_b5=0<br>ks_b5_bf=0<br>Clb5T=0<br>ks_n2=0<br>ks_n2_bf=0<br>Cln2=0	-	[2.4] max(ESP1(t)) < 0.1
CLB5-*db*Δ<br>Viable	kd_b5_20=0<br>kd_b5_20_i=0	-	[0.137] max(ORI(t)) < 40
CLB5-*db*Δ *sic*1Δ<br>Origin Relicensing<br>Problems	kd_b5_20=0<br>kd_b5_20_i=0<br>ks_ki*=0.125<br>ks_ki_swi5*=0.125<br>CKIT=0.2	[7.54] min(CLB2(t)+CLB5(t)) > 0.25	-
GAL-CLB5<br>Viable	MDT=150<br>f=0.48<br>ks_b5=0.04	[0.593] max(ORI(t)) < 40<br>[1] FLAG_UDNA(τ) >= 1<br>[1] FLAG_UDNA(τ) >= 1	[0.512] max(ORI(t)) < 40

GAL-CLB5 *sic*1Δ<br>Origin Relicensing Problems	MDT=150<br>f=0.48<br>ks_b5=0.04<br>ks_ki*=0.125<br>ks_ki_swi5*=0.125<br>CKIT=0.2	[0.605] max(ORI(t)) < 40	[0.477] max(ORI(t)) < 40
GAL-CLB5 *cdh*1Δ<br>Inviable	MDT=150<br>f=0.48<br>ks_b5=0.04<br>CDH1T=0<br>CDH1A=0	-	-
GAL-CLB5-*db*Δ<br>Origin Relicensing Problems	MDT=150<br>f=0.48<br>ks_b5=0.04<br>kd_b5_20=0<br>kd_b5_20_i=0	[0.599] max(ORI(t)) < 40	[15] max(DIV_COUNT(t)) > 5<br>[23.6] max(ORI(t)) < 40
*clb*1Δ *clb*2Δ<br>S/G2 Arrest	ks_b2=0<br>ks_b2_m1=0<br>Clb2T=0	-	[2.4] max(ESP1(t)) < 0.1
*clb*1Δ *clb*2Δ *clb*5Δ *clb*6Δ<br>G1 Arrest	ks_b2=0<br>ks_b2_m1=0<br>Clb2T=0<br>ks_b5=0<br>ks_b5_bf=0<br>Clb5T=0	-	[2.4] max(ESP1(t)) < 0.1
GAL-CLB2<br>Viable	MDT=150<br>f=0.48<br>ks_b2*=49.40	[0.0722] max($V(t)$) < 8<br>[12] max(DIV_COUNT(t)) > 5	[0.0788] max(ORI(t)) < 40<br>[4.12] FLAG_BUD(τ) >= 1<br>[6.82] FLAG_BUD(τ) >= 1<br>[6.78] FLAG_BUD(τ) >= 1<br>[6.44] FLAG_BUD(τ) >= 1
Multicopy *GAL*-CLB2<br>Telophase Arrest	MDT=150<br>f=0.48<br>ks_b2*=242.42	-	-
GAL-CLB2 *sic*1Δ<br>Telophase Arrest	MDT=150<br>f=0.48<br>ks_b2*=49.40<br>ks_ki*=0.125<br>ks_ki_swi5*=0.125<br>CKIT=0.2	-	-
GAL-CLB2 *cdh*1Δ<br>Telophase Arrest	MDT=150<br>f=0.48<br>ks_b2*=49.40<br>CDH1T=0<br>CDH1A=0	-	-
GAL-CLB2 *swi*5Δ<br>Telophase Arrest	MDT=150<br>f=0.48<br>ks_b2*=49.40<br>ks_swi5=0<br>ks_swi5_m1=0<br>SWI5T=0	-	-
CLB2-*db*Δ<br>Telophase Arrest	kd_b2_20=0<br>kd_b2_h1*=0.09<br>kd_b2_20_i=0	[1.48] ORI(τ) > 5	-
CLB2-*db*Δ in galactose<br>Telophase Arrest	kd_b2_20=0<br>kd_b2_h1*=0.09<br>kd_b2_20_i=0<br>MDT=150<br>f=0.48	[0.699] ORI(τ) > 5	-
CLB2-*db*Δ<br>*GAL-SIC*1<br>Viable	kd_b2_20=0<br>kd_b2_h1*=0.09<br>kd_b2_20_i=0<br>MDT=150<br>f=0.48<br>ks_ki*=33.33	-	-

CLB2-*db*Δ<br>multicopy $SIC1$<br>Viable	kd_b2_20=0<br>kd_b2_h1*=0.09<br>kd_b2_20_i=0<br>ks_ki*=65<br>ks_ki_swi5*=65	[2.31] max($V(t)$) $<$ 8<br>[15] max(DIV_COUNT(t)) $>$ 5<br>[3.03] max(ORI(t)) $<$ 40	[4.14] FLAG_BUD(τ) $>=$ 1<br>[3.7] FLAG_BUD(τ) $>=$ 1<br>[7.68] FLAG_SPC(τ) $>=$ 1<br>[3.86] FLAG_SPC(τ) $>=$ 1
CLB2-*db*Δ *clb*5Δ<br>*clb*6Δ<br>Telophase Arrest	kd_b2_20=0<br>kd_b2_h1*=0.09<br>kd_b2_20_i=0<br>ks_b5=0<br>ks_b5_bf=0<br>Clb5T=0	[2.51] ORI(τ) $>$ 5	-
CLB2-*db*Δ *clb*5Δ<br>*clb*6Δ in galactose<br>Viable	kd_b2_20=0<br>kd_b2_h1*=0.09<br>kd_b2_20_i=0<br>ks_b5=0<br>ks_b5_bf=0<br>Clb5T=0<br>MDT=150<br>f=0.48	[0.194] max($V(t)$) $<$ 8<br>[15] max(DIV_COUNT(t)) $>$ 5	[0.509] max($V(t)$) $<$ 8<br>[15] max(DIV_COUNT(t)) $>$ 5<br>[310] max(ORI(t)) $<$ 40<br>[6.91] FLAG_BUD(τ) $>=$ 1
GAL-CLB2-*db*Δ<br>Telophase Arrest	kd_b2_20=0<br>kd_b2_h1*=0.09<br>kd_b2_20_i=0<br>MDT=150<br>f=0.48<br>ks_b2*=49.40	-	-
$CLB1$ *clb*2Δ<br>Viable	ks_b2*=0.33<br>ks_b2_m1*=0.33	-	-
$CLB1$ *clb*2Δ *cdh*1Δ<br>Inviable	ks_b2*=0.33<br>ks_b2_m1*=0.33<br>CDH1T=0<br>CDH1A=0	-	[33.6] max($V(t)$) $>$ 20<br>[25] max(DIV_COUNT(t)) $<$ 3
$CLB1$ *clb*2Δ *pds*1Δ<br>Inviable	ks_b2*=0.33<br>ks_b2_m1*=0.33<br>ks_pds=0<br>PDS1T=0	-	[33.9] max($V(t)$) $>$ 20<br>[20] max(DIV_COUNT(t)) $<$ 3
*cdc*20-*ts*<br>Metaphase Arrest	ks_20=0<br>ks_20_m1=0<br>CDC20T=0<br>CDC20A_APCP=0	-	[0.825] max(ESP1(t)) $<$ 0.1
*clb*5Δ *clb*6Δ<br>*cdc*20Δ<br>Metaphase Arrest	ks_b5=0<br>ks_b5_bf=0<br>Clb5T=0<br>ks_20=0<br>ks_20_m1=0<br>CDC20T=0<br>CDC20A_APCP=0	-	[0.825] max(ESP1(t)) $<$ 0.1
*cdc*20Δ *pds*1Δ<br>Telophase Arrest	ks_20=0<br>ks_20_m1=0<br>CDC20T=0<br>CDC20A_APCP=0<br>ks_pds=0<br>PDS1T=0	[10] SPN(τ) $>$ 1	-
*clb*5Δ *clb*6Δ<br>*cdc*20Δ *pds*1Δ<br>Viable	ks_b5=0<br>ks_b5_bf=0<br>Clb5T=0<br>ks_20=0<br>ks_20_m1=0<br>CDC20T=0<br>CDC20A_APCP=0<br>ks_pds=0<br>PDS1T=0	[2.31] max($V(t)$) $<$ 8<br>[15] max(DIV_COUNT(t)) $>$ 5	-

CLB5-$db\Delta$ $cdc20\Delta$ $pds1\Delta$<br>Telophase Arrest	kd_b5_20=0<br>kd_b5_20_i=0<br>ks_20=0<br>ks_20_m1=0<br>CDC20T=0<br>CDC20A_APCP=0<br>ks_pds=0<br>PDS1T=0	[10] SPN(τ) > 1	-
CLB5-$db\Delta$ $pds1\Delta$<br>Viable	kd_b5_20=0<br>kd_b5_20_i=0<br>ks_pds=0<br>PDS1T=0	[2.31] max($V(t)$) < 8<br>[15] max(DIV_COUNT(t)) > 5	[0.0598] max(ORI(t)) < 40
*GAL-CDC*20<br>Mitotic Catastrophe	MDT=150<br>f=0.48<br>ks_20*=1666.67	[4] ESP1(τ) > 0.2	[4] ESP1(τ) > 0.2
*GALL-CDC*20 $sic1\Delta$ $cdh1\Delta$<br>Viable	MDT=150<br>f=0.48<br>ks_20*=1000<br>ks_ki*=0.125<br>ks_ki_swi5*=0.125<br>CKIT=0.2<br>CDH1T=0<br>CDH1A=0	-	[7.49] FLAG_BUD(τ) >= 1<br>[7.55] FLAG_BUD(τ) >= 1
*GALL-CDC*20 $sic1\Delta$ $cdc6\Delta$2-49 $cdh1\Delta$<br>Viable	MDT=150<br>f=0.48<br>ks_20*=1000<br>ks_ki=0<br>ks_ki_swi5=0<br>CKIT=0<br>CKIP=0<br>CDH1T=0<br>CDH1A=0	-	[7.46] FLAG_BUD(τ) >= 1<br>[7.53] FLAG_BUD(τ) >= 1
APC-A<br>Viable	ka_cp_b2=0	-	[5.49] max($V(t)$) < 8<br>[15] max(DIV_COUNT(t)) > 5<br>[604] max(ORI(t)) < 40
APC-A $sic1\Delta$<br>Viable	ka_cp_b2=0<br>ks_ki*=0.125<br>ks_ki_swi5*=0.125<br>CKIT=0.2	-	[5.49] max($V(t)$) < 8<br>[15] max(DIV_COUNT(t)) > 5<br>[604] max(ORI(t)) < 40
APC-A $sic1\Delta$ $cdc6\Delta$2-49<br>Viable	ka_cp_b2=0<br>ks_ki=0<br>ks_ki_swi5=0<br>CKIT=0<br>CKIP=0	-	[5.49] max($V(t)$) < 8<br>[15] max(DIV_COUNT(t)) > 5<br>[604] max(ORI(t)) < 40<br>[7.47] FLAG_BUD(τ) >= 1
APC-A $cdh1\Delta$<br>Telophase Arrest	ka_cp_b2=0<br>CDH1T=0<br>CDH1A=0	[3.24] ORI(τ) > 5	-
APC-A $cdh1\Delta$ in galactose<br>Telophase Arrest	ka_cp_b2=0<br>CDH1T=0<br>CDH1A=0<br>MDT=150<br>f=0.48	[1.63] ORI(τ) > 5	-
APC-A $cdh1\Delta$ *GAL-SIC*1<br>Viable	ka_cp_b2=0<br>CDH1T=0<br>CDH1A=0<br>MDT=150<br>f=0.48<br>ks_ki*=33.33	-	[0.172] max(ORI(t)) < 40<br>[3.62] FLAG_BUD(τ) >= 1<br>[4.59] FLAG_BUD(τ) >= 1<br>[3.81] FLAG_BUD(τ) >= 1
APC-A $cdh1\Delta$ multicopy *SIC*1<br>Viable	ka_cp_b2=0<br>CDH1T=0<br>CDH1A=0<br>ks_ki*=65<br>ks_ki_swi5*=65	-	[0.00166] max(ORI(t)) < 40<br>[5.32] FLAG_BUD(τ) >= 1<br>[5.85] FLAG_BUD(τ) >= 1<br>[7.44] FLAG_BUD(τ) >= 1<br>[1.3] FLAG_SPC(τ) >= 1

APC-A cdh1Δ multicopy *CDC20* Viable	ka_cp_b2=0<br>CDH1T=0<br>CDH1A=0<br>ks_20*=2<br>ks_20_m1*=2	[2.31] max($V(t)$) $<$ 8<br>[15] max(DIV_COUNT(t)) $>$ 5<br>[1.03] max(ORI(t)) $<$ 40	[4.11] FLAG_BUD(τ) >= 1<br>[8.47] FLAG_BUD(τ) >= 1<br>[8.4] FLAG_BUD(τ) >= 1<br>[8.15] FLAG_BUD(τ) >= 1
APC-A GAL-CLB2 Telophase Arrest	ka_cp_b2=0<br>MDT=150<br>f=0.48<br>ks_b2*=49.40	-	-
pds1Δ Viable	ks_pds=0<br>PDS1T=0	[2.31] max($V(t)$) $<$ 8<br>[15] max(DIV_COUNT(t)) $>$ 5	-
PDS1-dbΔ Inviable	kd_pds_20=0	-	-
GAL-PDS1-dbΔ Inviable	MDT=150<br>f=0.48<br>ks_pds*=3.33<br>kd_pds_20=0	-	-
esp1-ts Inviable	ESP1T=0	-	-
GAL-PDS1-dbΔ *esp1-ts* Inviable	MDT=150<br>f=0.48<br>ks_pds*=3.33<br>kd_pds_20=0<br>ESP1T=0	-	-
GAL-ESP1 *cdc20-ts* Inviable	MDT=150<br>f=0.48<br>ESP1T*=2<br>ks_20=0<br>ks_20_m1=0<br>CDC20T=0<br>CDC20A_APCP=0	-	-
ppxΔ Viable	PPXT=0<br>PPX=0	[2.31] max($V(t)$) $<$ 8<br>[15] max(DIV_COUNT(t)) $>$ 5	-
GAL-PPX Viable	MDT=150<br>f=0.48<br>PPXT*=2	-	-
tem1Δ Telophase Arrest	TEM1T=0<br>TEM1=0	[0.751] ORI(τ) $>$ 5<br>[1.66] SPN(τ) $>$ 1<br>[4] ESP1(τ) $>$ 0.2	-
net1-ts Viable	kas_net*=0.45	-	-
tem1Δ net1-ts Viable	TEM1T=0<br>TEM1=0<br>kas_net*=0.45	-	-
GAL-TEM1 Viable	TEM1T*=5	-	-
tem1-ts *GAL-CDC15* Viable	TEM1T=0<br>TEM1=0<br>MDT=150<br>f=0.48<br>CDC15T*=10	[1] FLAG_UDNA(τ) >= 1	-
tem1-ts multicopy *CDC14* Viable	TEM1T=0<br>TEM1=0<br>CDC14T*=2	-	-
Multicopy *CDC15* Viable	CDC15T*=20	-	-
tem1-ts multicopy *CDC15* Viable	TEM1T=0<br>TEM1=0<br>CDC15T*=20	-	-
net1-ts cdc20-ts Inviable	kas_net*=0.45<br>ks_20=0<br>ks_20_m1=0<br>CDC20T=0<br>CDC20A_APCP=0	-	-

cdc15Δ<br>Telophase Arrest	CDC15T=0	[0.718] ORI(τ) > 5<br>[1.52] SPN(τ) > 1<br>[4] ESP1(τ) > 0.2	[33.9] max($V(t)$) > 20<br>[20] max(DIV_COUNT(t)) < 3
cdc15Δ net1-ts<br>Viable	CDC15T=0<br>kas_net*=0.45	-	-
cdc15Δ net1-ts<br>*cdh1Δ*<br>Viable	CDC15T=0<br>kas_net*=0.45<br>CDH1T=0<br>CDH1A=0	[2.31] max($V(t)$) < 8<br>[15] max(DIV_COUNT(t)) > 5<br>[2.27] max(ORI(t)) < 40	[5.49] max($V(t)$) < 8<br>[15] max(DIV_COUNT(t)) > 5<br>[200] max(ORI(t)) < 40
cdc15-ts multicopy<br>*TEM1*<br>Telophase Arrest	CDC15T=0<br>TEM1T*=5	[0.718] ORI(τ) > 5<br>[1.52] SPN(τ) > 1<br>[4] ESP1(τ) > 0.2	[33.9] max($V(t)$) > 20<br>[20] max(DIV_COUNT(t)) < 3
cdc15-ts multicopy<br>*CDC14*<br>Viable	CDC15T=0<br>CDC14T*=2	-	-
TAB6-1<br>Viable	kas_net*=0.5	-	-
cdc15Δ TAB6-1<br>Viable	CDC15T=0<br>kas_net*=0.5	-	-
TAB6-1 clb5Δ<br>*clb6Δ*<br>G1 Arrest	kas_net*=0.5<br>ks_b5=0<br>ks_b5_bf=0<br>Clb5T=0	-	[2.4] max(ESP1(t)) < 0.1
TAB6-1 CLB1<br>*clb2Δ*<br>Viable	kas_net*=0.5<br>ks_b2*=0.33<br>ks_b2_m1*=0.33	-	[0.036] max(ORI(t)) < 40
cdc14-ts<br>Telophase Arrest	CDC14T=0	-	-
cdc14-ts sic1Δ<br>Telophase Arrest	CDC14T=0<br>ks_ki*=0.125<br>ks_ki_swi5*=0.125<br>CKIT=0.2	-	-
cdc14-ts cdh1Δ<br>Telophase Arrest	CDC14T=0<br>CDH1T=0<br>CDH1A=0	-	-
cdc14-ts<br>*GAL-SIC1*<br>Telophase Arrest	CDC14T=0<br>MDT=150<br>f=0.48<br>ks_ki*=33.33	[0.673] ORI(τ) > 5	-
cdc14-ts<br>*GAL-CLN2*<br>Telophase Arrest	CDC14T=0<br>MDT=150<br>f=0.48<br>ks_n2=0.15	-	-
GAL-NET1<br>Telophase Arrest	MDT=150<br>f=0.48<br>NET1T*=10.85	[11.6] max($V(t)$) > 20<br>[0.186] ORI(τ) > 5	[37.1] max($V(t)$) > 20<br>[15] max(DIV_COUNT(t)) < 3
GAL-CDC14<br>G1 Arrest	MDT=150<br>f=0.48<br>CDC14T*=7	-	[2.4] max(ESP1(t)) < 0.1<br>[67.9] max(ORI(t)) < 0.75
GAL-NET1<br>*GAL-CDC14*<br>Viable	MDT=150<br>f=0.48<br>CDC14T*=7<br>NET1T*=10.85	-	-

Supplementary Table 3: Search space and final fit value for each parameter in the model. In the study of Oguz et al. 2013 (ref. [2]), the search space was chosen based on a nominal estimate of each parameter value, given in column 2 below. Their fitting algorithm considered a uniform search space spanning up to ± 90% of these nominal values. For example, for the parameter gamma with an estimated value of 1, their search space ranged from 0.1 to 1.9. We took a similar approach, and centered our search space on the same nominal values as ref. [2], but considered a larger search space: A log uniform space spanning ± 2 orders of magnitude from the nominal values. For example, for the parameter gamma, we considered values ranging from 0.01 to 100. Our methodology would be expected to find a good fit even if the nominal values were inaccurate (by up to 2 orders of magnitude).

Param	Center of log uniform search space	Fit value
gamma	1.00	2.22
gammaki	10.0	12.9
gammacp	1.00	1.34
gammatem	1.00	0.369
sig	10.0	9.63
signet	10.0	1.52
ks_n3	1.50	1.11
Jn3	6.00	4.27
Dn3	1.00	0.732
kd_n3	3.00	0.794
ks_k2	0.135	0.0553
kd_k2	2.50	3.01
kdp_i5	1.00	1.22
kdp_i5_14	0.100	0.195
kp_i5	0.100	0.0275
kp_i5_n3	6.00	6.10
kp_i5_k2	6.00	23.7
kp_i5_n2	15.0	2.97
kp_i5_b5	0.100	0.0422
kdp_bf	1.00	2.93
kp_bf_b2	8.00	9.36
ks_n2	0.00	1.00e-8
ks_n2_bf	0.500	0.996
kd_n2	0.250	0.0320
ks_ki	0.0120	6.63e-3
ks_ki_swi5	0.120	0.0890
kd_ki	0.0100	0.0524
kd_kip	2.00	0.899
kp_ki_e	1.00	0.650
e_ki_n3	2.50	1.05
e_ki_k2	0.500	0.397
e_ki_n2	1.00	19.5
e_ki_b5	3.00	2.39
e_ki_b2	4.00	3.12
kdp_ki	1.00	0.836
kdp_ki_14	7.00	1.11
ks_b5	1.60e-3	5.38e-4
ks_b5_bf	0.0100	0.0178
kd_b5	0.0100	0.0556
kd_b5_20	0.160	0.0445
ks_b2	2.00e-3	7.62e-3
ks_b2_m1	0.150	0.0310
kd_b2	3.00e-3	2.98e-3
kd_b2_20	0.0900	0.136
kd_b2_h1	0.400	0.662
ks_bud_e	0.200	0.287
e_bud_n3	0.0500	7.80e-3
e_bud_n2	0.250	1.12
e_bud_b5	1.00	3.00
e_bud_b2	0.100	1.89
kd_bud	0.0600	0.0590
ks_spn	0.100	0.0743
kd_spn	0.0600	0.0384
Jspn	0.140	0.809
ks_ori_e	2.00	1.90
e_ori_b5	0.900	5.04

e_ori_b2	0.450	0.124
kd_ori	0.0600	0.0817
ks_swi5	5.00e-3	5.58e-3
ks_swi5_m1	0.0800	0.0389
kd_swi5	0.0800	0.0420
ka_swi5_14	2.00	1.41
ki_swi5_b2	0.0500	0.0280
ka_m1_b2	10.0	4.65
ki_m1	1.00	3.39
ks_20	6.00e-3	0.0221
ks_20_m1	0.600	0.354
kd_20	0.300	0.124
ka_20	0.100	0.0104
kd_b5_20_i	0.0120	4.98e-3
kd_b2_20_i	0.0320	0.0374
ki_20_ori	8.00	5.04
ka_cp_b2	1.00	0.334
ki_cp	1.00	0.210
ka_h1	1.00	0.241
ka_h1_14	7.50	32.2
ki_h1	0.100	0.144
ki_h1_e	1.00	0.215
e_h1_n3	0.250	3.75
e_h1_n2	0.700	1.56
e_h1_b5	7.00	9.73
e_h1_b2	8.00	2.35
kdp_net	1.00	0.106
kdp_net_14	0.100	6.63e-3
kdp_net_px	10.0	83.3
kp_net	0.100	0.556
kp_net_b2	1.00	1.50
kp_net_en	10.0	6.88
kp_net_15	0.0500	8.81e-3
ka_px	1.00	0.0550
ki_px	0.100	0.119
ki_px_p1	3.00	6.69
ks_pds	0.0300	0.0467
kd_pds	0.0500	0.0144
kd_pds_20	3.00	3.04
ka_15	0.100	0.709
ka_15_14	5.00	7.38
ki_15	1.00	0.894
ki_15_b2	1.00	2.16
ka_tem	0.100	0.0848
ka_tem_lo	2.00	3.84
ka_tem_p1	0.100	0.0638
ki_tem	1.00	0.323
ki_tem_px	2.00	1.92
ks_lo	0.0100	0.0450
ks_lo_m1	0.100	0.0113
kd_lo	0.0100	4.83e-3
kd_lo_h1	0.0300	0.139
ka_lo	0.100	0.0232
ka_lo_b2	3.00	1.11
ki_lo	1.00	0.965
kas_net	1.00	5.61
WHI5T	3.00	2.10
SBFT	1.00	0.468
MCM1T	1.00	0.282
APCPT	25.0	45.7
CDH1T	1.00	0.808
NET1T	3.00	6.40
CDC14T	2.00	6.23
PPXT	1.00	0.866
ESP1T	0.500	0.264
CDC15T	1.00	1.02

TEM1T	1.00	1.29
kd_pds_20_i	0.300	0.125
Initial Conditions		
V_0	1.25	0.460
$Cln3_0$	0.180	0.725
$Bck2_0$	0.0660	0.0209
$WHI5dep_0$	2.02	1.77
$SBFdep_0$	0.990	0.678
$Cln2_0$	0.100	0.0626
$CKIT_0$	1.05	0.0556
$CKIP_0$	0.100	0.0350
$Clb5T_0$	0.290	0.0519
$Clb2T_0$	0.100	0.0148
BUD_0	0.0500	0.0240
ORI_0	0.100	0.0657
SPN_0	0.110	0.149
$SWI5T_0$	0.210	0.122
$CDC20T_0$	0.0200	0.0803
$CDC20A\_APCP_0$	0.100	0.260
$APCP_0$	0.100	1.48
$CDH1A_0$	1.00	2.75
$NET1deP_0$	2.70	2.47
PPX_0	0.870	0.323
$PDS1T_0$	0.240	0.236
$CDC15_0$	0.100	0.646
$TEM1_0$	0.100	0.0713
$POLOT_0$	0.670	0.0927
$POLOA_0$	0.100	0.0997
$CDC20A\_APC_0$	0.100	0.0535
Phase Parameters		
Param	Uniform search space	Fit value
ϕ_α	500-650	571
ϕ_{cdc15}	500-650	537
ϕ_{cdc28}	500-650	599
Constants		
Constant	Value	
MDT	100	
f	0.4	

Supplementary References

[1] Spellman, P. T. *et al*. Comprehensive identification of cell cycle-regulated genes of the yeast *Saccharomyces cerevisiae* by microarray hybridization. *Mol. Biol. Cell* **9**, 3273–3297 (1998).

[2] Oguz, C. *et al.* Optimization and model reduction in the high dimensional parameter space of a budding yeast cell cycle model. *BMC Syst. Biol.* **7**, 53 (2013).
