# Supplementary material for: Using both qualitative and quantitative data in parameter identification for systems biology models
Source: Nat Commun. 2018 Sep 25;9:3901. doi: 10.1038/s41467-018-06439-z (PMC6156341; doi:10.1038/s41467-018-06439-z)
Supplement: Supplementary file 3 — Description of Additional Supplementary Files [file 41467_2018_6439_MOESM3_ESM.pdf]

Description of Additional Supplementary Files

Supplementary Software 1

Description: Python and C++ code associated with the Raf and yeast cell cycle models used in this study, and SBML implementations of both models.
